# A Unidirectional Soft Dielectric Elastomer Actuator Enabled by Built-In Honeycomb Metastructures

**DOI:** 10.3390/polym12030619

**Published:** 2020-03-09

**Authors:** Kun Liu, Shitong Chen, Feifei Chen, Xiangyang Zhu

**Affiliations:** 1 State Key Laboratory of Mechanical System and Vibration, School of Mechanical Engineering, Shanghai Jiao Tong University, Shanghai 200240, China; kianliu@sjtu.edu.cn (K.L.); stonehot@sjtu.edu.cn (S.C.); mexyzhu@sjtu.edu.cn (X.Z.); 2 Robotics Institute, School of Mechanical Engineering, Shanghai Jiao Tong University, Shanghai 200240, China

**Keywords:** dielectric elastomer actuators, honeycomb, metastructure, 3D printing

## Abstract

Dielectric elastomer actuators (DEAs) are able to undergo large deformation in response to external electric stimuli and have been widely used to drive soft robotic systems, due to their advantageous attributes comparable to biological muscles. However, due to their isotropic material properties, it has been challenging to generate programmable actuation, e.g., along a predefined direction. In this paper, we provide an innovative solution to this problem by harnessing honeycomb metastructures to program the mechanical behavior of dielectric elastomers. The honeycomb metastructures not only provide mechanical prestretches for DEAs but, more importantly, transfer the areal expansion of DEAs into directional deformation, by virtue of the inherent anisotropy. To achieve uniaxial actuation and maximize its magnitude, we develop a finite element analysis model and study how the prestretch ratios and the honeycomb structuring tailor the voltage-induced deformation. We also provide an easy-to-implement and scalable fabrication solution by directly printing honeycomb lattices made of thermoplastic polyurethane on dielectric membranes with natural bonding. The preliminary experiments demonstrate that our designed DEA is able to undergo unidirectional motion, with the nominal strain reaching up to 15.8%. Our work represents an initial step to program deformation of DEAs with metastructures.

## 1. Introduction

Soft robotics, an emerging multidisciplinary field, has been receiving increasing attention in the past decade, due to the flexibility and adaptability offered by soft materials to interact with unstructured environments [1,2,3,4,5,6]. Soft actuators, typically made of compliant materials that are responsive to external physical stimuli, play a key role in enabling the functionalities of soft robots. Various actuation technologies has been developed, such as pneumatic or hydraulic actuators [7,8,9], shape memory polymers [10,11,12], ionic polymer-metal composites [13] and so on. Among them, dielectric elastomer actuators (DEAs) are a promising alternative because of their advantages such as fast response, high energy density and low cost [14,15,16,17,18]. A dielectric elastomer (DE) is typically a rubbery membrane, coated with compliant electrodes on its two surfaces. Its actuation principle is as follows: when an electric field is applied to a DE membrane, the Maxwell stress will be induced to squeeze the membrane in thickness and lead the membrane to expand in area. The simplicity of DEAs in terms of the structure and working principle permits direct energy transformation between electric energy and mechanical energy with high efficiency and large frequency bandwidth. With these distinguished properties, various soft DEA-based machines and robots have been developed, ranging from tactile displays [19,20,21] and grippers [22,23,24] to bioinspired locomotive robots [2,25,26] and flying microrobots [27].

When using DEAs to devise reconfigurable structures or robots, a fundamental issue is how to achieve desired deformations upon electric activation. For instance, confined motions along a specific direction may be in high demand for a soft DEA-based robot that works in a congested environment [2]. In a general sense, since DEs are homogeneous isotropic materials, it requires inclusion of anisotropy in terms of the actuator configuration or the electric activation to generate user-defined deformation. For example, Huang et al. [28] and Lu et al. [29] applied unidirectional dead loads to mechanically prestretch DEAs to produce uniaxial voltage-induced deformation, while the actuators were constrained in the orthogonal direction by attaching stiff fibers. However, it is rather complex and time-consuming to align fibers and affix them onto DE membranes.

An alternative solution is to use distributed electric stimuli to program the deformation of DEAs. Chen et al. [30] and Wang et al. [31] recently developed spatially varying electric fields via a topology optimization approach to maximize the directional actuation of the end-effector of DEAs, with applications in triggering shape-morphing of planar sheets into three dimensional configurations [32]. However, they focused on the motion of only some points of interest in the DE membrane and thus cannot ensure directional motion of the whole actuator. Besides, rigid mechanisms were used to prestretch the DE membranes, which can hardly be used for soft robots. Hajiesmaili and Clarke [33] achieved reconfigurable shape-morphing of stacked DEs, with gradient electric fields varying in the thickness direction. However, the proposed method is intuitive and can only generate simple deformation patterns. Therefore, it can be concluded that despite the works mentioned above, programming of DEAs’ deformation is still not fully addressed in a general sense.

**Figure 1:**
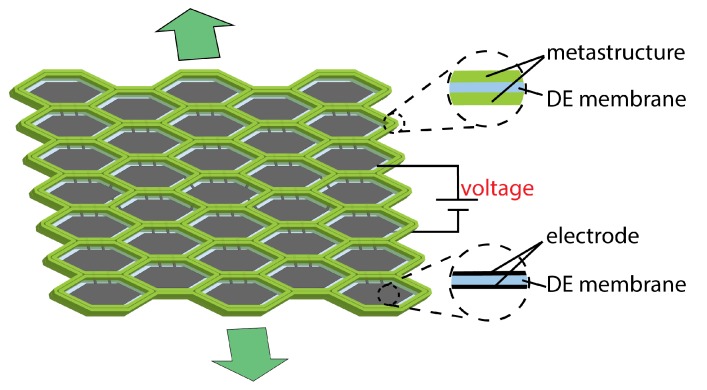
Schematic of a DE membrane coupled with honeycomb metastructures. When a high voltage is applied, the actuator outputs a unidirectional motion. The two insets illustrate the sections of the DE membrane sandwiched between metastructures and between electrodes, respectively.

Considering the fact that a DEA typically needs a support structure to provide some level of prestress in order to enlarge the actuation range and postpone material failures such as electric breakdown, in this paper, we focus on design of the support structure to enable directional deformations of DEAs. Metastructures usually produce considerable change in geometry in response to external loads, making them an ideal option for programming shape-morphing [34,35], e.g., novel structures with negative Poisson’s ratio [36,37,38]. However, to our best knowledge, few works have applied metastructures to DEAs. In this paper, we use honeycomb metastructures as support structures and harness their structural anisotropy to achieve unidirectional deformation of a DEA. As shown in Figure 1, an array of honeycomb cells featured with strong anisotropy stand on a DE membrane. By simply tuning the geometry parameter of the honeycomb lattices, we may obtain a family of support structures with a wide range of geometric and mechanical properties.

To provide insight into design of the proposed actuator, we develop a finite element analysis model and study the effect of the geometry parameters of the honeycomb and the prestretch ratios of the DE membrane on the actuation performance. We find that a unidirectional actuation can be achieved when the honeycomb is of a particular shape. To fabricate the actuator prototype, we first pattern the electrode cells on the DE membrane and similar to [39], we directly print the honeycomb lattices made of thermoplastic polyurethane (TPU) on the membrane. It is found that the solidified TPU can be firmly bonded onto the DE membrane. We further carry out preliminary experiments to evaluate the voltage-induced deformation of the actuator, and the results show that the uniaxial strain reaches up to 15.8% at 7.5 kV, while the strain in its orthogonal direction is effectively suppressed. This finding validates that metastructures can be effective enablers for DEAs to generate programmable deformations.

In summary, in this paper, we develop a unidirectional DEA enabled by built-in honeycomb metastructures. The contributions of our work lie in the following aspects. First, we propose an innovative solution to programming deformations of DEAs, by using anisotropic honeycomb lattices as support structures. Second, we offer an easily accessible and scalable fabrication solution to building periodic metastructures on DE membranes by 3D printing. The printer (Ultimaker) and materials (TPU) are commercially available and affordable. Finally, on the other way, we provide a new actuation technology for metastructures which are typically mechanically loaded.

The remainder of this paper is organized as follows. Section 2 describes the materials we used and the corresponding fabrication process. Section 3 introduces the hyperelastic material model of DEAs and finite element analysis results in the commercial software ABAQUS. Section 4 demonstrates and discusses the experimental results. Section 5 concludes the paper and envisages the future work.

## 2. Materials and Fabrication

We sandwich a DE membrane between two symmetric layers of honeycomb lattices to construct a planar actuator. The honeycomb cells each are painted with compliant electrodes and all the electrodes are interconnected so that only one high voltage signal is needed for activation. In this section, we will introduce the materials we adopt for DEAs and honeycombs, and dictate the fabrication process in detail.

### 2.1. Materials Selection

Acrylic elastomers VHB 4910/4905 (3M company) and Wacker films are widely used as commercial DEs. We select VHB 4910 because it is very sticky and thus can be naturally bonded with honeycomb metastructures made of TPU. Since DEs are soft and deformable, the electrodes are expected to be compliant as well. Carbon grease is naturally fluidic and thus is widely used as compliant electrodes for DEAs. However, the fluidic nature of carbon grease also causes several issues. First, carbon grease is prone to pollute the metastructures. Besides, the predefined electrode pattern may be easily disrupted when printing the metastructures on the DE membrane. The nozzle of the printer may also be stained. Therefore, we use curable electrodes provided by Wacker company [40] that are in solidified state at room temperature and can be firmly attached to VHB without causing contamination.

The honeycomb lattices are printed by fused deposition modeling (FDM). Abundant open source materials for FDM printers are commercially available, among which TPU is one kind of the materials widely used for printing flexible structure because of its relatively low tensile modulus (<100 MPa) [41]. Meanwhile, elongation at yield of TPU reaches up to 55% [41], and thus it can withstand large deformation.

### 2.2. Fabrication

**Figure 2:**
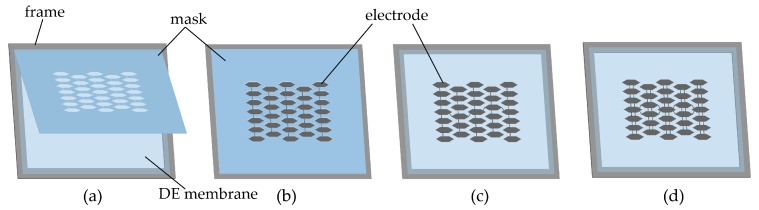
Fabrication process of patterning curable electrodes on a DE membrane.

The whole fabrication process contains two parts: patterning electrodes on a DE membrane and then printing metastructures on the membrane. As shown in Figure 2, the electrode patterning process is as follows: (1) prestretch the VHB 4910 membrane by $\lambda_{1}\times \lambda_{2}$; (2) fix the prestretched membrane onto a rigid frame; (3) fabricate a mask made of release paper by laser cutting and attach it onto the DE membrane (Figure 2a); (4) paste curable electrodes onto the DE membrane by Trek trans-printer (Figure 2b); (5) remove the mask and cure the electrode in the oven for half an hour under 70 ${}^{\circ }$C (Figure 2c); (6) repeat the steps above to trans-print the electrode on the other side of the DE membrane (Figure 2d). To ensure that the electrodes of the honeycomb cells are interconnected with each other, the leads are painted on the two sides of the DE membrane, and they are well spaced out to avoid possible electric breakdown on the leads.

**Figure 3:**
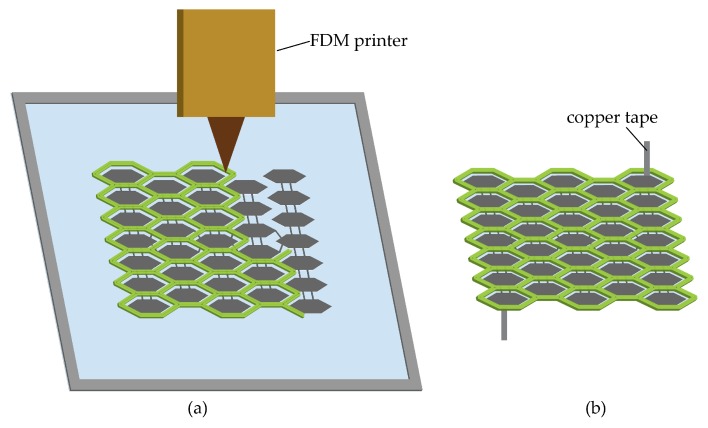
(**a**) Print metastructures on a DE membrane with an FDM printer. (**b**) Release the actuator prototype from the substrate membrane and connect copper tapes to it.

After the electrodes are cured, we start printing the honeycomb metastructures on the two sides of the DE membrane using an FDM printer (Ultimaker), as shown in Figure 3a. After printing, the DE membrane is cut off along the periphery of the metastructures. Upon release, the prestretched membrane with built-in honeycombs will shrink to some extent until reaching an equilibrium state as a minimum energy structure as shown in Figure 3b. Copper tapes connect the actuator to a high voltage power.

## 3. Model and Simulation

To predict the electromechanical performance of the proposed DEA, in this section, we carry out finite element analysis and study the effect of the honeycomb geometry parameters on the resulting voltage-induced deformation of the DEA. In this way, we obtain a feasible design that gives rise to unidirectional motions. In the analysis, the high voltage activation is simulated by a spatial electric field, with the electrode substances omitted.

### 3.1. Modeling of DEs

The Neo-Hookean model is widely used to describe the hyperelasticity of DEs, with the free energy density *W* being function of the mechanical stretches

(1) $$W=\frac{\mu }{2}\left(\lambda_{1}^{2}+\lambda_{2}^{2}+\lambda_{3}^{2}-3\right)$$

where μ is the shear modulus of the elastomer, and $\lambda_{1}$, $\lambda_{2}$ and $\lambda_{3}$ are the principal stretches with subscripts 1, 2 denoting the *x*, *y* direction in the membrane plane and 3 the normal direction of the membrane. DEs are typically taken to be incompressible, that is, $\lambda_{1}\lambda_{2}\lambda_{3}=1$.

According to the theory of ideal dielectric elastomers by Suo and Zhao [42,43], the stress and the stretch follow the relation:

(2) $$\sigma_{1}+\epsilon E^{2}=\lambda_{1}\frac{\partial W}{\partial \lambda_{1}}$$

(3) $$\sigma_{2}+\epsilon E^{2}=\lambda_{2}\frac{\partial W}{\partial \lambda_{2}}$$

where $\sigma_{1}$, $\sigma_{2}$ are the principal Cauchy stresses in the plane, *E* represents the electric field applied to the dielectric membrane and ϵ is the permittivity independent of the deformation. The material properties of VHB 4910 are shown in Table 1. Based on the constitutive equations, we use a user-defined material subroutine to simulate the voltage-induced response of DE membranes.

### 3.2. Finite Element Analysis

#### 3.2.1. Setting

**Figure 4:**
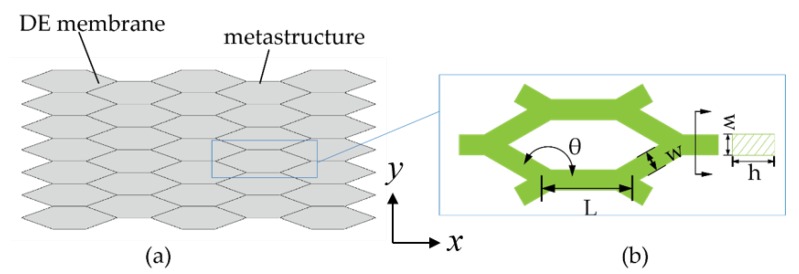
The CAD model in ABAQUS: (**a**) assembly of the DE membrane and honeycombs; (**b**) the geometry parameters of each honeycomb cell.

We carry out finite element analysis in the commercial software ABAQUS to study how the design parameters will tailor the actuation performance of the DEA. Since it is a two dimensional problem, the two separate metastructure parts on the two sides of the DE membrane are substituted by an integrated part. The CAD model is shown in Figure 4a. The honeycomb cells are modeled by beam element (b31) for efficient computation. The honeycomb metastructures are modeled by linear elastic materials, with material properties as listed in Table 2. It is noted that ideally, the flexural modulus of elasticity is equivalent to the tensile modulus (Young’s modulus) of elasticity for rectangular beam. The interface between the DE membrane and metastructures is defined as tie interaction in ABAQUS along the edges of the cells. The applied voltage will directly determine the induced Maxwell stress in the subroutine, and thus affect the resulting deformation of the actuator.

We carry out finite element analysis and study the effect of the parameters involved on the actuation performance that is captured by the axial voltage-induced strains. Specifically, we define the nominal axial strains $\varepsilon_{x}$ and $\varepsilon_{y}$ as follows

(4) $$\varepsilon_{x}=\frac{u_{x}}{L_{1}}$$

(5) $$\varepsilon_{y}=\frac{u_{y}}{L_{2}}$$

where $u_{x}$ and $u_{y}$ denote the voltage-induced displacements along the *x* and *y* directions, respectively, and $L_{1}$ and $L_{2}$ denote the actuator dimensions along the *x* and *y* directions before activation, respectively.

Within each honeycomb cell, the main geometry parameters include the height *h*, the width of the beam *w*, the length of each beam *L*, and the interior angle of the cells θ as shown in Figure 4b. The simulation results show the tendency that a smaller width *w* and a larger length *L* of the beam would give rise to a larger actuation strain. It is worth mentioning that, however, a beam too narrow cannot guarantee secure bonding between TPU and the DE membrane, and a beam too thin or too long may lead the whole actuator to buckle into three dimensional configurations which is undesired in this work. In practice, we determine the parameters *h*, *w* and *L* based on experimental observations, and their values are then fixed, as listed in Table 2.

We investigate the resulting axial actuations through finite element analysis, and find that the prestretch ratios $\lambda_{1}$ and $\lambda_{2}$ and the angle θ in the honeycomb cell play significant roles. For convenience and clarity, in the following we will demonstrate two groups of parameters: (1) keep the honeycomb angle and change the prestretch ratios; (2) keep the prestretch ratios and change the honeycomb angle.

#### 3.2.2. Effect of Prestretches: $\lambda_{1}$ and $\lambda_{2}$

We vary the prestretch ratios and investigate the resulting axial strains, as listed in Table 3 and Table 4, with the honeycomb angle being fixed at $\theta =90^{\circ }$ and $\theta =150^{\circ }$, respectively. It is observed that the prestretch ratios $\lambda_{1}$ and $\lambda_{2}$ both have important impact on the resulting axial strains. Because the honeycomb cell is anisotropic, even biaxial prestretches lead to different axial strains (see case 3 in Table 3 and Table 4). Specifically, for $\theta =90^{\circ }$, it is found that regardless of the prestretch ratios, the resulting strains in the plane are limited (<9%), and they are comparable in the two directions. When the honeycomb angle increases to $\theta =150^{\circ }$, the metastructures become highly anisotropic. Specifically, when $\lambda_{1}=4.0$ and $\lambda_{2}=2.5$, the actuator is equipped with strong anisotropy in that the *y*-axis strain (31.41%) is much larger than the *x*-axis strain (−3.32%), and therefore these prestretch ratios are selected for the final prototyping.

#### 3.2.3. Effect of Honeycomb Angle θ

**Figure 5:**
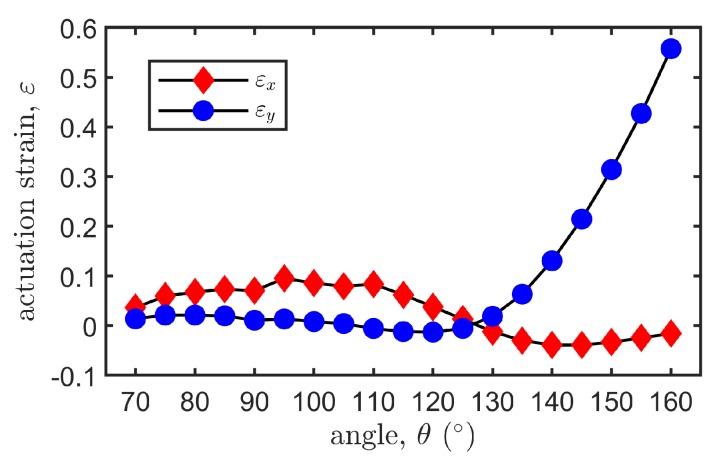
Voltage-induced axial strains $\varepsilon_{x}$ and $\varepsilon_{y}$ with different θ under $\lambda_{1}=4.0$, $\lambda_{2}=2.5$.

It should be noticed that when the honeycomb angle θ is less than 70${}^{\circ }$ or lager than 160${}^{\circ }$, the structures will collapse themselves after release. Figure 5 shows the relation between the voltage-induced axial strains $\varepsilon_{x},\varepsilon_{y}$ and the honeycomb angle θ with the prestretch ratios being fixed at $\lambda_{1}=4.0,\lambda_{2}=2.5$. It is observed that the axial strains both vary with the honeycomb angle, which can be positive or negative. Specifically, the *x*-axis strain remains at a relatively low level and fluctuates around zero, bounded within 10% tensile strain or compressive strain, while the *y*-axis strain may vary significantly with the honeycomb angle. When $\theta >120^{\circ }$, the *y*-axis strain increases monotonically. Considering the fact that the honeycomb cell becomes slender for a large angle, which demands more precise fabrication, we set the honeycomb angle to be $\theta =150^{\circ }$.

### 3.3. Final Design for Prototyping

**Figure 6:**
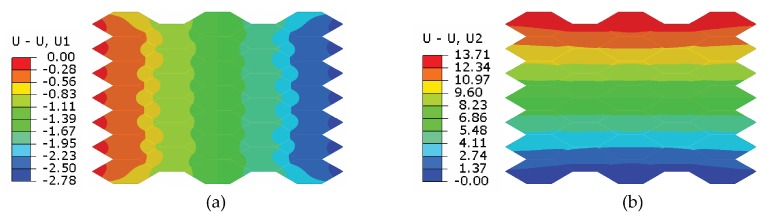
The actuation displacements (unit: mm) in (**a**) *x* and (**b**) *y* directions at 7.5 kV.

After parameter studies, we obtain a proper configuration ($\theta =150^{\circ }$ and prestretch ratios $\lambda_{1}=4.0$, $\lambda_{2}=2.5$) with obvious unidirectional actuation. The voltage-induced deformation is shown in Figure 6. The initial size of the actuator before being powered is 83.8 mm × 43.6 mm. When the applied voltage reaches 7.5 kV, the maximal displacement along *y* direction is 13.71 mm, resulting in an actuation strain of 31.44%, while the actuator shrinks along *x* direction by 2.78 mm, resulting in a compressive actuation strain of 3.32%.

## 4. Experimental Section

### 4.1. Setup

**Figure 7:**
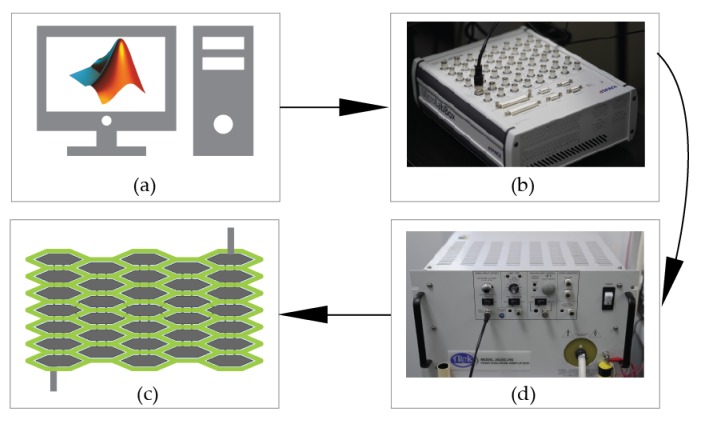
The experimental setup: (**a**) Simulink; (**b**) dSPACE; (**d**) amplifier; (**c**) actuator prototype.

The experimental setup is shown in Figure 7. The actuator is powered with a high voltage provided by the amplifier (20/20B-HS, Trek, Inc., Medina, NY, USA). The amplifier with a fixed gain of 2000 is connected to the DAC port of the dSPACE (dSPACE-DS1202). The dSPACE outputs analog signals through its DAC port and runs the complied model generated by the Simulink. The deformation of the actuator is recorded by a camera (Sony FE90F2.8) and the software AE (Adobe Effect CC2018) is used to track the points of interest on the actuator.

### 4.2. Results

**Figure 8:**
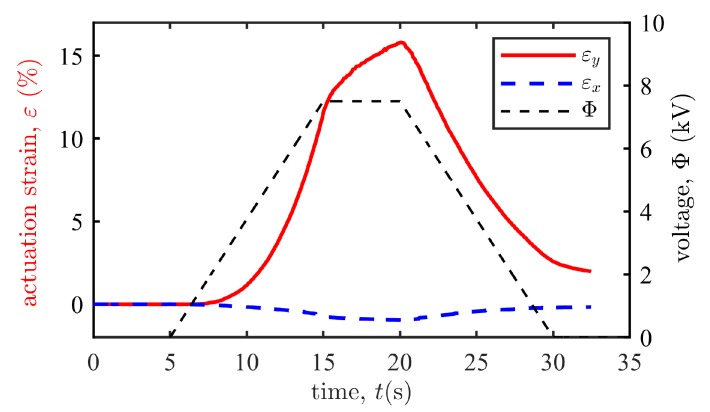
The actuation strains $\varepsilon_{x}$ and $\varepsilon_{y}$ vary with the trapezoid input voltage.

**Figure 9:**
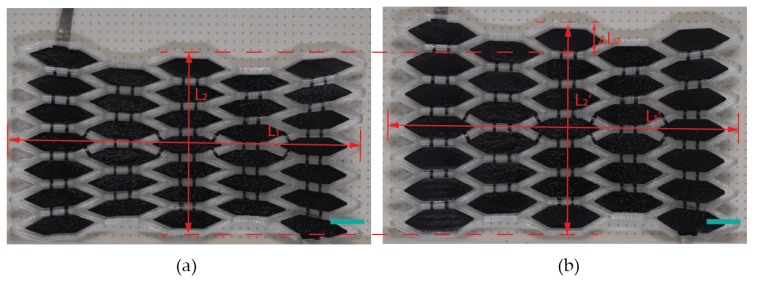
The actuator configurations: (**a**) the released state before activation and (**b**) the most deformed state at 7.5 kV. The scale bar is 8 mm.

To evaluate the performance of the actuator, a trapezoid voltage Φ (black dashed line in Figure 8) is applied to the actuator and leads to its planar configurational change, with the initial state and the most deformed state shown in Figure 9. We use the middle points along the four edges in Figure 9 to capture the axial displacement of the actuator. The deformation process is recorded and the actuation strains that vary with the applied voltage are calculated and plotted in Figure 8. As expected, the *x*-axis strain $\varepsilon_{x}$ fluctuates at a low level, while the *y*-axis strain $\varepsilon_{y}$ changes significantly, resulting in a unidirectional motion of the actuator (see the Video S1 in the Supplementary Materials). When the applied voltage is 7.5 kV, the resulting axial strains are $\varepsilon_{x}=-0.97\%$ and $\varepsilon_{y}=15.8\%$, with the measured dimensions in Figure 9 being $L_{1}=82.59$ mm, $L_{2}=43.16$ mm, $L_{1}^{\prime }=81.79$ mm, and $L_{2}^{\prime }=49.99$ mm. When the applied voltage increases further, the DE membrane may suffer material failures such as loss of tension and electric breakdown.

**Figure 10:**
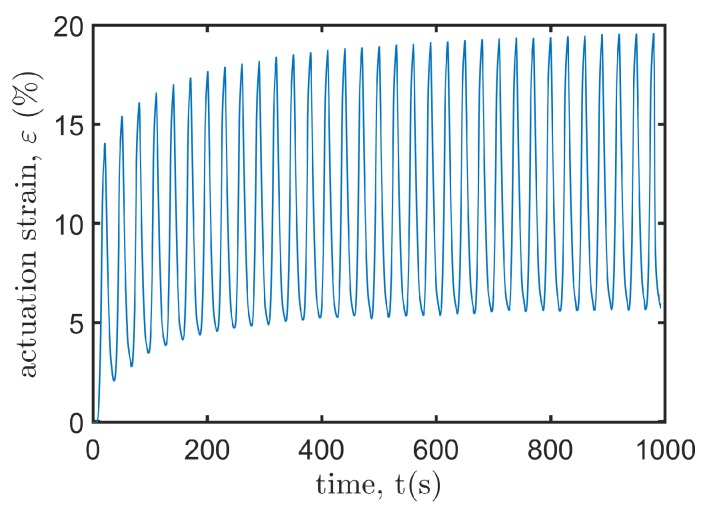
The actuation strain $\varepsilon_{x}$ varies with the cyclic trapezoid input voltage.

It is observed from Figure 8 that the *y*-axis strain grows nonlinearly with the applied voltage, partly because of the geometric nonlinearity of the actuator, and partly because the voltage-induced Maxwell stress that deforms the DE membrane is proportional to the square of the voltage. To investigate the dynamic response of the actuator, we apply periodic trapezoid voltages to the actuator. As shown in Figure 10, the resulting axial strain $\varepsilon_{x}$ oscillates with time. In the first few cycles, we observe remarkable viscoelastic creep which captures the drift effect of the resulting deformation of the DE material when the applied voltage keeps unchanged [44]. The axial strain tends to be stable gradually (discrepancies between peak values are less than 0.3% after 14 cycles). The decrement of the stable strokes compared with the first actuation cycle is less than 0.5%, which manifests good repeatability of cyclic actuations in producing stable strokes. Besides, hysteresis is also observed by noting the discrepancy of the voltage-induced displacements during the loading and unloading processes. This phenomenon is also due to the well-known viscoelasticity of DEs [18].

### 4.3. Discussion

There exists considerable discrepancy between the simulation and experimental results. This discrepancy may be caused by at least two reasons. First, the experimental effective area of the electric field is smaller than the simulation counterpart. In the simulation, the whole DE membrane is subject to the applied electric field. However, this is not the case for the real experiments where the electrodes are separated in cells. The cells are spaced out to make room for printing the TPU on the DE membrane, and they are interconnected by leads. Considering the accuracy of positioning between the electrode cells and the metastructures, a gap of 3 mm is retained. Second, the honeycomb lattices are modeled by beam elements in the finite element analysis, and thus the width of the beam is neglected in the CAD model. To this end, the simulation result has somehow overestimated the actuation strains by neglecting the component contributed by the metastructures when calculating the actuator length. In particular, this error will grow pronounced with the increase of the honeycomb angle.

To address the discrepancy above and improve the actuation performance, advanced microfabrication technologies [45] may be used to seamlessly pattern the electrodes in the printed TPU honeycomb lattices. In this way, more areas of the DE membrane will be activated and a larger axial actuation strain can be expected.

Another significant concern is that the requirement for high voltage could hinder the proposed actuator for practical applications. It is noticed that, recently, Ji et al. have largely addressed this issue by fabricating stacked ultrathin DE membranes, powered by a low voltage of 450 V [46]. They clarified that below 500 V, there exists a vast selection of low-mass, miniature, commodity surface-mount components, enabling complex kilohertz voltage sources weighing only tens or hundreds of milligrams to be integrated into soft robotic systems. It is hoped that with the advance of more accessible and scalable microfabrication technologies, more DEA-based practical applications can be explored.

## 5. Conclusions and Future Outlook

In this paper, we propose a novel DEA with metastructures built-in that achieves a directional actuation strain of 15.8%. The honeycomb metastructures not only provide prestretches for the DE membrane, but also equip the DE with high anisotropy to output uniaxial actuations. The prestretch ratios and the honeycomb angle are delicately designed, through finite element analysis, in order to improve the actuation along the prescribed direction. We also develop an integrated fabrication process by directly printing metastructures made of TPU on the DE membrane with secure bonding after solidification. Our work paves the way for scenarios of using metastructures to program motions of DEAs.

In the future, we aim to achieve more programmable deformation patterns of the DEAs, by coordinating the distributed honeycomb angles and modulating the distributed voltages. For example, the applied voltages can be switched on and off cell-wise to change the global deformation behavior. We also hope to develop soft locomotive robots based on the programmed DEAs.

## Figures and Tables

**Table 1:** Material properties of VHB 4910.

Original Thickness	Shear Modulus	Dielectric Constant	Breakdown Strength
1 mm	45 kPa	$4.0\times 10^{-11}$ F/m	∼100 MV/m

**Table 2:** Material and geometry parameters of honeycomb metastructures.

Flexural Modulus	Poisson’s Ratio	Beam Length *L*	Beam Width *w*	Beam Height *h*
78 MPa	0.36	8 mm	0.8 mm	2 mm

**Table 3:** The actuation strains with different prestretches for $\theta =90^{\circ },\Phi$ = 7.5 kV.

Case	$\lambda_{1}$	$\lambda_{2}$	$\varepsilon_{x}$ (%)	$\varepsilon_{y}$ (%)
1	4.0	2.5	7.02	1.09
2	4.0	2.0	5.57	1.13
3	3.2	3.2	5.71	0.66
4	3.0	3.0	4.87	0.58
5	2.5	4.0	8.40	0.69
6	2.0	4.0	7.56	0.58

**Table 4:** The actuation strains with different prestretches for $\theta =150^{\circ },\Phi$ = 7.5 kV.

Case	$\lambda_{1}$	$\lambda_{2}$	$\varepsilon_{x}$ (%)	$\varepsilon_{y}$ (%)
1	4.0	2.5	−3.32	31.41
2	4.0	2.0	−3.28	23.77
3	3.2	3.2	−1.71	30.97
4	3.0	3.0	−1.44	26.59
5	2.5	4.0	−0.62	23.27
6	2.0	4.0	−0.27	14.45

## Supplementary Materials

The following are available online at https://www.mdpi.com/2073-4360/12/3/619/s1.
